# Method Development for Simultaneously Determining Indomethacin and Nicotinamide in New Combination in Oral Dosage Formulations and Co-Amorphous Systems Using Three UV Spectrophotometric Techniques

**DOI:** 10.1155/2024/2035824

**Published:** 2024-02-20

**Authors:** Nazira Sarkis, Abdulkader Sawan

**Affiliations:** Department of Analytical and Food Chemistry, Faculty of Pharmacy, University of Aleppo, Aleppo, Syria

## Abstract

This research aims to develop methods for simultaneously determining indomethacin (IND) and nicotinamide (NCT) in binary mixtures, immediate-release capsules, sustained-release capsules, and co-amorphous systems, which were designed in 2021 to improve the solubility, dissolution rate, and stability of the amorphous state of indomethacin. Moreover, this new combination may have also other possible medical benefits. Therefore, there is a need to have simple, sensitive, and precise developed methods for simultaneous quantification analysis of IND/NCT in several different ratios. Three UV-spectrophotometry techniques were deployed: zero-crossing point in the second-order derivative, dual-wavelength in the first-order derivative, and ratio subtraction coupled with spectrum subtraction. The limit of detection and the limit of quantifications (LOD and LOQ) for IND were 0.41 and 1.25, 0.55 and 1.66, and 0.53 and 1.62 *μ*g/mL, respectively, while for NCT were 0.53 and 1.59, 0.38 and 1.14, and 0.36 and 1.08 *μ*g/mL, respectively. All methods were linear at least in the range of 2.5–40.0 *μ*g/mL. All proposed methods were validated according to ICH guidelines and their application on the dosage formulations was carried out. Finally, the proposed methods were compared to a reference method for each IND and NCT, and no significant statistical variance was found.

## 1. Introduction

**Figure 1:**
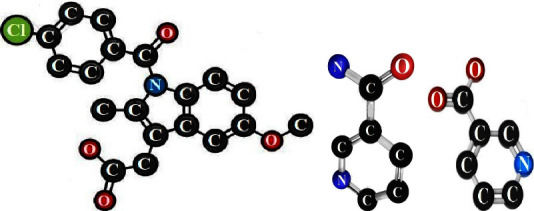
The chemical structures of indomethacin (a), nicotinamide (b), and nicotinic acid (c).

Indomethacin (IND) is one of the nonsteroidal anti-inflammatory drugs (NSAIDs) and a nonselective inhibitor of COX-1 and COX-2 with antipyretic and analgesic effects. IND is an indole-acetic acid derivative. Its full name is 2-[1(4-chlorobenzoyl)-5-methoxy-2-methylindol-3-yl] acetic acid with a molecular formula of C_18_H_16_ClNO_4_ as shown in Figure 1(a). It has a molecular weight of 357.08 g/mol. It is an odorless pale-yellow crystalline substance [1].

Nicotinamide (NCT), often known as niacinamide, is 3-pyridine carboxamide as shown in Figure 1(b) [2]. It is an amide form of nicotinic acid or niacin as shown in Figure 1(c). It is also colorless crystals or white crystalline powder. NCT weighs 122.12 g/mol at the molecular level. Additionally, it is known as vitamin B3, one of the hydrophilic B vitamins [3]. NCT may be added to multivitamins products and supplementary oral dosage formulations. It is also found in many dermal preparations, such as solutions, creams, gels, and serums, which are used for skin care and many skin conditions due to its renewal and anti-inflammation properties [4]. Different studies showed that NCT has a mild to moderate anti-inflammation effect that can be combined with anti-inflammatory agents for improved therapy [5–7]. NCT was found to be a hydrotropic solubilizing agent. Hydrotropy is a phenomenon when a solute enhances the solubility of another poorly soluble solute in water and aqueous solutions [8, 9]. Lastly, NCT or its derivative 1-methyl nicotinamide may also have antacid and protective effects to reduce the risk of gastric ulcers related to NSAID intake or stress [10–12].

IND can be delivered using various routes and formulations ranging from oral 25-50 mg immediate-release, 75–100 mg sustained-release capsules, and 25 mg/5 mL suspensions to rectal 50–100 mg suppositories [13]. It can be found alone or with other active ingredients such as paracetamol in ParinCare™ oral capsules or vitamin B1 (thiamine) in local pharmaceutical products, including Indovit® and Indobina® oral capsules. IND, like any other NSAIDs, may cause stomach aches and many patients use antacids to protect the stomach. Orally, IND is fully absorbed through the gastrointestinal tract and has virtually 100% bioavailability [14]. However, due to its poor aqueous solubility, it is classified as class II in the biopharmaceutical classification system (BCS), i.e., it has low solubility and high permeability. This may limit or delay absorption causing a possible decrease in bioavailability. Therefore, there are different methods used to improve the dissolution rate and solubility of class II drugs such as IND. Physicochemical characteristics of drugs can be changed by using crystal engineering techniques such as salt formation, solvate (or hydrate), amorphous forms, and more recently co-crystal and co-amorphous systems, which are common ways to do this for many NSAIDs and other drugs. The co-amorphous system does extra benefit in increasing the stability of the amorphous forms along with increasing solubility and dissolution rate [15, 16].

NCT was used in many studies as an acceptable carrier or coformer with other drugs in co-amorphous drug-drug systems such as atorvastatin and valsartan [15, 17]. In 2021, researchers published a study (Fael and Demirel, 2021) where they suggested and designed the IND and NCT binary combination formula in the form of a co-amorphous drug-drug system, which was prepared for oral administration [18]. The previous work achieved two progressions. First, this co-amorphous system succeeded in increasing the solubility and dissolution rate of IND in the gastrointestinal tract and in limiting the recrystallization of IND to improve the stability of its amorphous form. Second, this new combination itself has promising therapeutical advantages from decreasing the risk of gastric ulcers caused by IND [10, 11] to building synergism in order to increase pain relief depending on the suggested neuroprotective and anti-inflammation effect of NCT [5, 19, 20]. This new suggested formula of IND-NCT could be the first IND-NCT combination that has not been marketed worldwide yet. According to Fael and Demirel, 2021, this IND-NCT co-amorphous mixture was combined by mixing their powders and applying heating then using quench cooling with liquid nitrogen to finally create the co-amorphous system. This was carried out without adding any other ingredient to their mixture. Hence, it is possible to simultaneously estimate this binary mixture using UV spectrophotometric techniques.

In this work, UV spectroscopic techniques were developed, validated, and deployed to simultaneously determine the combination of IND and NCT in the binary mixtures and in the IND dosage formulations that were spiked with NCT with the same IND : NCT molar ratios (M : M) of 1 : 10, 1 : 5, 1 : 3, 1 : 2, 1 : 1, and 1 : 0.5. These ratios were suggested and tested in the previous work of Fael and Demirel, 2021. In this study, a weight/volume ratio (*μ*g/mL) has been used. Thus, after being approximately converted depending on their molecular weights, mass ratios of 1 : 3, 1 : 2, 1 : 1, 1 : 0.5, 1 : 0.33, and 1 : 0.17, respectively, were used. There are many UV spectrophotometric techniques, including a pharmacopeial method for NCT [21–23] in addition to other methods such as high-performance liquid chromatography (HPLC), including a pharmacopeial method for IND [24–26] and electrochemical techniques [27, 28] to determine NCT or IND in one-component dosage formulations as well as in combinations with other active ingredients. Although there are already developed UV spectrophotometric methods to analyze, only IND in the existence of NCT in the same solution was used in the procedure of analysis, and NCT was used as a hydrotropic solubilizing agent to increase IND aqueous solubility. Usually, this was intended to use an eco-friendly solution capable of dissolving IND using NCT as a hydrotrope in high concentrations like 0.5–2.0 M. In other words, NCT was not a second ingredient with IND in its dosage formulations. These previous methods estimate IND concentration using the specific range of UV spectrum (300–350 nm), where there is only absorption of IND and zero absorption of NCT, while the whole NCT range of absorption interfered with the signal of IND [9, 29]. Our proposed analysis methods are the first ones to simultaneously determine both IND and NCT.

In this paper, few analytical UV spectrophotometric methods have been developed and validated to simultaneously determine both IND and NCT in their mixtures and spiked dosage formulations without prior separation or processing. The results have been shown and discussed. The first and second-order derivative spectra have been deployed using zero-crossing point and dual-wavelength techniques, in addition to ratio subtraction and spectrum subtraction techniques. In total, three UV spectrophotometric methods were developed. These methods have been proven to be accurate, precise, fast, simple, and eco-friendly for many combinations and multicomponent dosage formulations without using any hazardous solvents or invasive materials [30–35]. Additionally, more advanced UV spectrophotometric methods have been developed initially from these reported ones deploying further mathematical processing [36–38].

NCT is freely soluble in water, ethanol, and methanol, while IND tends to be practically insoluble in water. However, IND is sparingly soluble in methanol and ethanol, which were used to dissolve IND and NCT [21, 23]. Additionally, ethanol has a low impact on the environment and a high greenness index. Also, ethanol could be considered a renewable solvent used in many green and sustainable analytical methods and procedures [39, 40]. Thus, ethanol is used as a solvent in the present suggested methods.

## 2. Materials and Methods

### 2.1. Instruments

The main instrument was T80+ UV-visible (PG instruments UK), a spectrophotometer device that was coupled with a computer and dedicated software. This device uses 1-centimeter-width quartz cells. Other instruments include an analytical balance (Sartorius, model 2474, Germany), an ultrasonic bath (Power sonic, model 405, Korea), a porcelain mortar, a centrifuge device (90-1 Centrifuge, Shanghai Surgical Instruments Factory, China), volumetric flasks, and pipettes.

### 2.2. Solvents and Chemicals

Substances used in this work included indomethacin powder with 99.5% purity (BDH Laboratory Supplies, England), nicotinamide powder with 99% purity (BDH Laboratory Supplies, England), and absolute ethanol of analytical grade (Halley Medical/Eurolab, United Kingdom). The tested dosage formulations include Arthacin®, immediate-release (IR) capsules, which contain 50 mg of IND (Oshar Pharma, Syria), Indomed®, and sustained-release (SR) capsules (SR), which contain 75 mg of IND (Medico Labs, Syria).

### 2.3. Preparation of Standard Solutions

#### 2.3.1. Standard Solution of Each Indomethacin and Nicotinamide

After weighing an equivalent amount to 62.5 mg of pure IND, it was transferred into a 20 mL flask and diluted with ethanol to obtain a standard stock solution of IND with a concentration of 3125 *μ*g/mL. Then, 2 mL was pipetted out to a 25 mL flask to obtain a stock solution of IND with a concentration of 250 *μ*g/mL. Finally, nine volumes of 0.1, 0.2, 0.4, 0.6, 0.8, 1.0, 1.2, 1.6, and 2.0 mL were pipetted out of the stock solution to nine 10 mL flasks and diluted with ethanol to prepare a set of standard solutions of IND with concentrations of 2.5, 5.0, 10.0, 15.0, 20.0, 25.0, 30.0, 40.0, and 50.0 *μ*g/mL, respectively. The same procedure was carried out to obtain NCT standard solution.

#### 2.3.2. Preparation of Standard Mixture of Nicotinamide and Indomethacin

Binary mixtures of IND and NCT were prepared following the same steps demonstrated in the previous Section 2.3.1. However, the 10 mL flasks of the final standard solutions were prepared from the previous 25 mL flasks of both IND and NCT stock solutions. These spiked mixtures with added NCT were prepared in ratios of 1 : 3, 1 : 2, 1 : 1, 1 : 0.5, 1 : 0.33, and 1 : 0.17 (IND : NCT), equivalent to the molar ratios suggested by the designers of the IND/NCT co-amorphous formula (Fael and Demirel, 2021). For either IND or NCT, additional concentrations such as 3.75, 7.50, 22.50, and 45.00 *μ*g/mL were prepared to obtain the previous ratios.

### 2.4. Preparation of the Mixture from Dosage Formulations

#### 2.4.1. From Immediate-Release (IR) Capsules Formulation

Arthacin®, an oral capsule dosage formulation, is produced by Osher Pharma Co. It is labeled to contain 50 mg of powdered IND in each hard gelatin capsule. Its labeled excipients in the leaflet were colloidal silicon dioxide, magnesium stearate, microcrystalline cellulose, powdered cellulose, sodium lauryl sulfate, and sodium starch glycolate.

Ten capsules were emptied and the powder was weighed. Four quantities of the powder, each of which containing 50 mg of IND, were weighed and transferred to 25 mL flasks separately. One of them will be analyzed after sonicating, diluting, and adding standard additions to determine IND in the presence of the excipients without NCT and to compare the developed methods with a reference method. The other three had NCT added to them in three ratios: 1 : 3, 1 : 1, and 1 : 0.33 (IND : NCT). After adding about 20 mL of ethanol to each of them, they were sonicated for 10 minutes. Then, ethanol was added to the mark. After that, they were transferred to tubes to be centrifuged for 15 minutes. Then, 0.5, 1.0, and 1.5 mL were transferred from the previous 20 mL flasks to other three 20 mL flasks to reach IND concentrations of 50, 100, and 150 *μ*g/mL and NCT concentrations of 150, 100, and 50 *μ*g/mL, and to preserve the ratios of 1 : 3, 1 : 1, and 1 : 0.33 (IND : NCT), respectively. From each of them, 1 mL was pipetted out to four 10 mL flasks. 50, 100, and 150% standard additions of IND and NCT were added to three of them from the pure stock solution flasks of each of IND and NCT from Section 2.3.1. The solutions were then ready to be scanned and analyzed by UV spectrophotometer. Final IND concentrations were between 5 and 15 *μ*g/mL without counting standard additions according to the labeled amount of IND in the capsules.

#### 2.4.2. Sustained-Release (SR) Capsules Formulation

Indomed®, an oral capsule formulation, is produced by Medico Pharma Labs. It is labeled to contain 75 mg of IND pellets, which is a combination of two types (immediate-release and delayed-release pellets) in each hard gelatin capsule. Its labeled excipients in the leaflet were corn starch, hydroxyl propyl cellulose, ethyl cellulose, lactose, sucrose, and titanium oxide.

The same procedures of the IR were carried out here except for some changes. First, capsules of this formulation contain pellets instead of powder. A mortar was used to triturate the pellets into powder after weighing an equivalent of 100 mg of IND. Second, three ratios of 1 : 1, 1 : 0.33, and 1 : 0.17 (IND : NCT) were prepared instead. After centrifuging, 1.0, 1.5, and 1.5 mL were transferred from the previous 20 mL flasks to other three 20 mL flasks to reach IND concentrations of 200, 300, and 300 *μ*g/mL and NCT concentrations of 200, 100, and 50 *μ*g/mL. Finally, from each of them, 0.5 mL was pipetted out to four 10 mL flasks before adding the standard additions (0, 50, 100, and 150%). The solutions were then ready to be scanned and analyzed by a UV spectrophotometer. Final IND concentrations were 10 or 15 *μ*g/mL without counting standard additions, according to the labeled amount of IND in the capsules.

### 2.5. UV Spectrophotometry Methods

Zero-order UV spectra of IND and NCT show IND's overlapping over the whole NCT absorption region. Three methods were found to simultaneously and quantitatively determine both of them.

#### 2.5.1. Zero-Crossing Point in Derivative Spectrophotometry Methods (ZCD_1_ and ZCD_2_)

First and second-order derivation (D_1_ and D_2_) of spectra of IND, NCT, and their mixture were created. One zero-crossing point of IND in the first derivation was found at 223.5 nm where NCT can be determined without any interference with IND or the excipients of its dosage formulations. However, after further examination and validation and because the absorptivity of both of them is high at this wavelength relative to the other region, it was not possible to simultaneously determine the mixtures probably with some ratios such as 1 : 3, 1 : 0.33, and 1 : 0.17, which made one of the components always reach the limit of the linearity range. Therefore, another zero-crossing point has to be found. In the second derivation, another one was found at 276.5 nm, where NCT can be determined without any interference from IND or the excipients of its dosage formulations. IND has a peak at 318 nm and there is no NCT signal at this wavelength, making it suitable for determining IND.

#### 2.5.2. Dual-Wavelength in First-Order Derivative Spectrophotometry Method (DWD_1_)

This method depends on locating two wavelengths *λ*_1_ and *λ*_2_, where the absorptivity (*ε*) of one component is the same at both of them (*ε*_1_ = *ε*_2_), and the second component has different absorptivity between the two wavelengths (*ε*_1_ ≠ *ε*_2_). The difference in the signal values of the two wavelengths (Δ*λ*) is only dependent on the concentration of the second component (C). A regression equation can be created between C and Δ *λ* as shown in this equation (Δ *λ* = a C + b) where “a” is the slope and “b” is the intercept with the *Y* axis. In the first-order derivative spectrum, 264.5 and 275.0 nm were found to have equal absorptivity for the NCT spectrum (*ε*_NCT1_ = *ε*_NCT2_). Therefore, they can be used to determine IND. 254.0 and 266.0 nm were found to have equal absorptivity for the IND spectrum (*ε*_IND1_ = *ε*_IND2_). Thus, they can be used to determine NCT.

#### 2.5.3. Ratio Subtraction and Spectrum Subtraction Methods (RS and SS)

RS and SS are fingerprint techniques that allow us to extract a spectrum that is almost identical to one of the pure solutions of each component of the binary mixture. Zero-order Spectrum of a mixture can be divided by the spectrum of the pure standard solution (divisor) of one component that has a region within the spectrum with only absorption of this component and zero absorption of the other one. The resulting spectrum is called a ratio spectrum and has special characteristics. First, it has a constant region (or plateau region) that represents zero absorption region by the second component. Second, any two points of it, except for the constant region, have different values dependent on the second component only. After creating the ratio spectrum, the value of the constant region is subtracted from the spectrum. The resulting spectrum is then multiplied by the divisor spectrum to create a new resolved spectrum representing the second component and theoretically has a fingerprint spectrum that is almost identical to the one obtained by a pure solution of the second component, whose concentration could be determined at its peak *λ*_max_ without any effect caused by the first component.

To determine the first component, we can subtract the newly resolved spectrum, which represents the second component, from the spectrum of the mixture to obtain a fingerprint spectrum that is almost identical to the one obtained by a pure solution of the first component, whose concentration could be determined at any proper wavelength without any effect caused by the second component.

IND is the one that has a noninterfering region. Thus, it has been chosen as a divisor. Choosing the best concertation of the standard solution as a divisor can be performed by finding a concentration with a minimum average absolute difference (AAD), which is simply the average of differences between the value of the constant in the ratio spectra created from a series of different concentration of the mixture and the value created from a series of pure standard solutions with same concentrations. The best concentration is the one that gives the minimum AAD. As a result, 15 *μ*g/mL of IND was the most suitable concentration to pick as a divisor. The concentration of NCT was determined at *λ*_max_ at 261.5 nm, while 268.0 nm was picked for IND determination.

Spectral ratio factor (SRF) was calculated to measure the similarity between the resolved spectra and the corresponding pure standard ones and to ensure the purity of the resolved spectra. SRF is usually a tool to measure the purity of a substance and it is used in this study to test the specificity of RS and SS by testing the purity of the resolved spectra and comparing them to the pure standard ones. Three wavelengths are chosen: *λ*_max_, *λ*_1_, and *λ*_2_ for both the resolved spectra and standard ones [41].

(1) $$\begin{array}{c} R_{1}=\frac{{A\lambda }_{\max}}{{A\lambda }_{1}},\\ R_{2}=\frac{{A\lambda }_{\max}}{{A\lambda }_{2}}, \end{array}$$

for the resolved spectrum:

(2) $$\begin{array}{c} F_{x}=\frac{R_{x1}}{R_{x2}}=\frac{{A\lambda }_{x2}}{{A\lambda }_{x1}}, \end{array}$$

for the standard spectrum:

(3) $$\begin{array}{c} F_{\text{st}}=\frac{R_{\text{st}1}}{R_{\text{st}2}}=\frac{{A\lambda }_{\text{st}2}}{{A\lambda }_{\text{st}1}},\\ \text{SRF}=\frac{F_{x}}{F_{\text{st}}}. \end{array}$$

The closer SRF gets to 1, the higher the purity and similarity the resolved spectrum has with the standard one. This indicates high specificity.

## 3. Results and Discussion

### 3.1. Finding the Optimal Wavelength for the Proposed Methods

**Figure 2:**
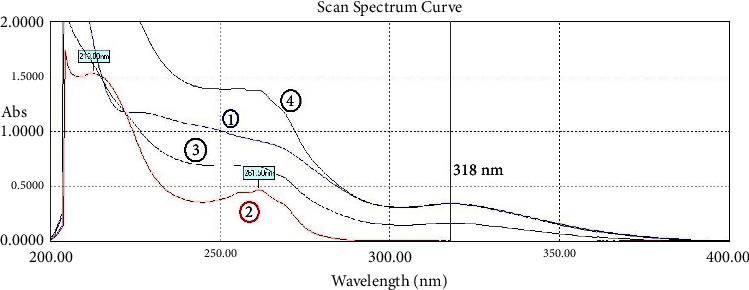
The zero-order UV absorption spectra of (1) indomethacin (20 *μ*g/mL) with 318 nm peak, (2) nicotinamide (20 *μ*g/mL) with 261.5 nm peak, (3) their binary mixture (NCT 10 *μ*g/mL + IND 10 *μ*g/mL), and (4) the same mixture with 20 *μ*g/mL of both.

**Figure 3:**
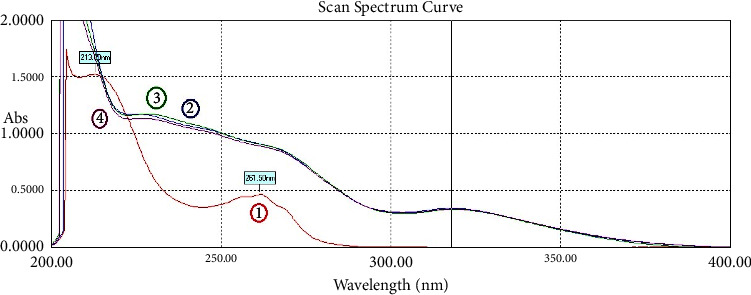
The zero-order UV absorption spectra of (1) nicotinamide 20 *μ*g/mL (red), (2) indomethacin 20 *μ*g/mL (blue), and the dosage formulations of indomethacin with the same concentration ((3) immediate-release capsules (green), and (4) sustained-release ones (purple)).

Pure solutions of IND, NCT, and their mixtures have been scanned with the spectrophotometer. The zero-order spectra are shown in Figure 2. Spectra of the IND dosage formulations are shown in Figure 3, which demonstrate that the excipients of both dosage formulations of IND did not have much effect on the spectrum of IND, especially in the region between 250 and 400 nm.

**Figure 4:**
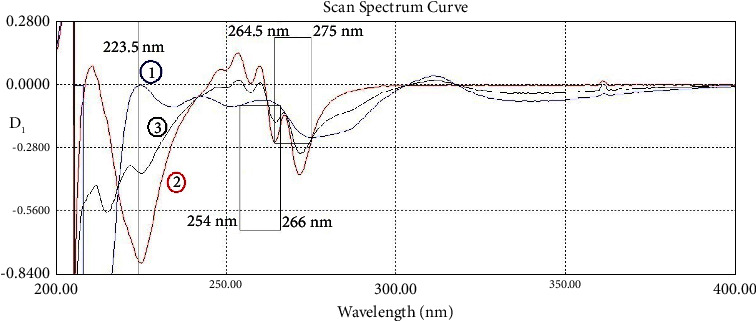
The first-order derivative spectra of (1) indomethacin 20 *μ*g/mL (blue), (2) nicotinamide 20 *μ*g/mL (red), and (3) their mixture 10 + 10 *μ*g/mL (black). Scaling factor ×10.

The first-order derivative, which was amplified 10 times (scaling factor/amplifying coefficient was 10), was created for the dual-wavelength method (DWD_1_). Dual-wavelength points were found to be 264.5 and 275.0 nm to determine IND (equal absorptivity for NCT) and 254.0 and 266.0 nm to determine NCT (equal absorptivity for IND) as shown in Figure 4. Also in the same figure, the zero-crossing point of IND at 223.5 nm is shown.

**Figure 5:**
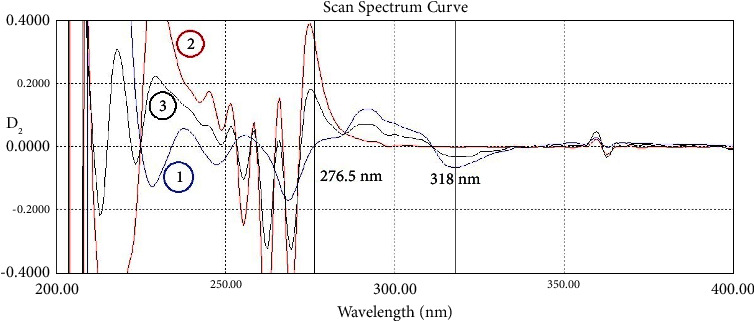
The second-order derivative spectra of (1) indomethacin 20 *μ*g/mL (blue), (2) nicotinamide spectrum 20 *μ*g/mL (red), and (3) their mixture 10 + 10 *μ*g/mL (black). Scaling factor ×40.

In the second-order derivative, which was amplified 40 times (scaling factor/amplifying coefficient was ×40), the best zero-crossing point of IND to determine NCT was found to be 276.5 nm. The best point to determine IND in D_2_ was the IND peak at 318.0 nm, where there was no absorption of NCT. This zero-crossing method in D_2_ (ZCD_2_) is shown in Figure 5.

**Figure 6:**
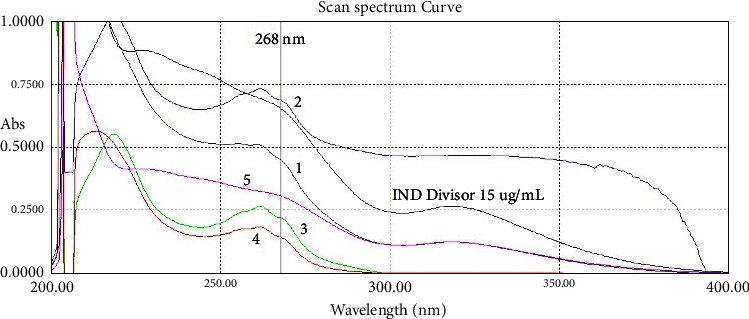
IND divisor spectrum with 15 *μ*g/mL (black). (1) The mixture spectrum 7.5 + 7.5 *μ*g/mL (indigo), (2) the ratio spectrum of the mixture (blue), (3) the ratio spectrum after constant subtraction (green), (4) the resolved spectrum of NCT (red), and (5) the resolved spectrum of IND (violet).

For the third method (RS and SS), a ratio spectrum was created, and the application of the method to retrieve the NCT and IND pure spectra from their mixture spectrum was carried out. The selected wavelength to determine IND is 268 nm and the one to determine NCT is 261.5 nm at its *λ*_max_. This is shown in Figure 6. To calculate the SRF used in specificity tests, two wavelengths (*λ*_1_ and *λ*_2_), which have the same absorptivity in the standard solutions, were picked. *λ*_1_ and *λ*_2_ are 295.0 and 318.0 nm, respectively, for IND, and they are 256.0 and 263.5 nm, respectively, for NCT.

### 3.2. Method Validation

#### 3.2.1. Linearity

The spectra of the IND and NCT series were scanned, and the linearity was tested for the three methods. Additionally, the determination coefficient (*r*^2^), the limit of detection (LOD), and the limit of quantification (LOQ) were calculated. The findings are displayed in Table 1. Additionally, the results of the system suitability test were placed in the last row of Table 1. System suitability was carried out by measuring six repetitions with a concentration of 20 *μ*g/mL for each IND and NCT and calculating the RSD% of them for each method. The results demonstrate low LOD-LOQ for both IND and NCT, acceptable determination coefficients *r*^2^, and a wide linearity range. Data were gathered and handled in compliance with ICH guidelines, and the proposed methods were verified in concurrence with ICH criteria with the following tests [42]. For NCT linearity range, it may have a higher upper limit than 50–60 *μ*g/mL in the zero-order spectrum but because whenever NCT exists with IND in any of the studied ratios, their mixtures get out of linearity above that previous concentration, which is true with IND too. IND linearity range may also have a higher upper limit than 50 *μ*g/mL by itself at its 318.0 nm peak but it is not possible to determine NCT when reaching above this concentration.

#### 3.2.2. Accuracy Test

It was performed for IND and NCT for the three methods. Three repetitions for each of the six concentrations were analyzed.  Table 2 shows the means and the relative standard deviations (RSD%) of the total 18 samples of NCT and IND. Six concentrations from the linearity range instead of three were studied because some ratios of the mixture have one of the components being only useable in either the upper or lower half of the linearity range. Due to this issue with these ratios, six concentrations spread through the whole linearity range were studied in this test. The proposed methods appear to achieve the validation criteria in the accuracy test according to ICH in a wide range of concentrations.

#### 3.2.3. Precision Test

It was achieved using three repetitions for each of the three concentrations (7.5, 15, and 22.5 *μ*g/mL for IND and 7.5, 15, and 30 *μ*g/mL for NCT) for each method to test the intraday repeatability precision. The same number of samples were analyzed once during each of the following two days to test the intermediate interday precision with a total of 27 samples. RSD% values of the nine samples each day are shown in Table 3 beside the total RSD% of all the 27 samples. The proposed methods appear to achieve the validation criteria in the precision test according to ICH.

#### 3.2.4. Specificity Test

**Figure 7:**
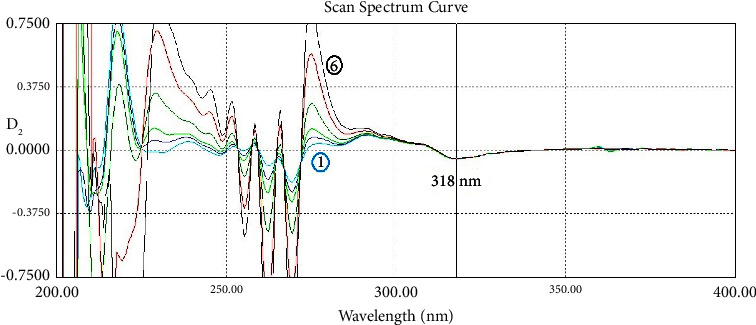
The second-order derivative spectra show a specificity test of different mixtures with 6 ratios containing constant indomethacin 15 *μ*g/mL and series of nicotinamide 2.5 (light blue), 5, 7.5, 15, 30, and 45 (black) *μ*g/mL. They give the same signal value at 318 nm.

**Figure 8:**
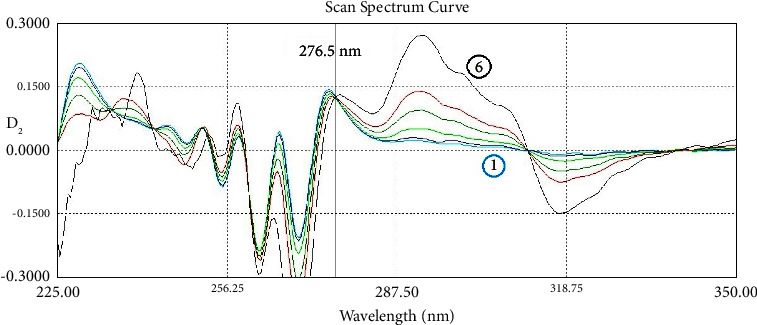
The second-order derivative spectra show a specificity test of 6 different mixtures with 6 ratios containing constant nicotinamide 7.5 *μ*g/mL and series of indomethacin (1) 2.5 (light blue), 3.75, 7.5, 15, 22.5, and (6) 45 (black) *μ*g/mL at 276.5 nm. They give the same signal value at 276.5 nm.

**Figure 9:**
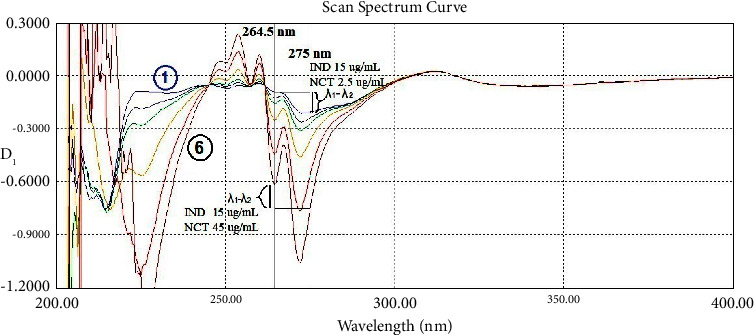
The first-order derivative spectra show a specificity test of different mixtures with 6 ratios containing constant indomethacin 15 *μ*g/mL and series of nicotinamide 2.5 (blue), 5, 7.5, 15, 30, and 45 (black) *μ*g/mL at 264.5 and 275 nm as *λ*_1_ and *λ*_2_. All of them have the same value of *λ*_1_-*λ*_2_.

**Figure 10:**
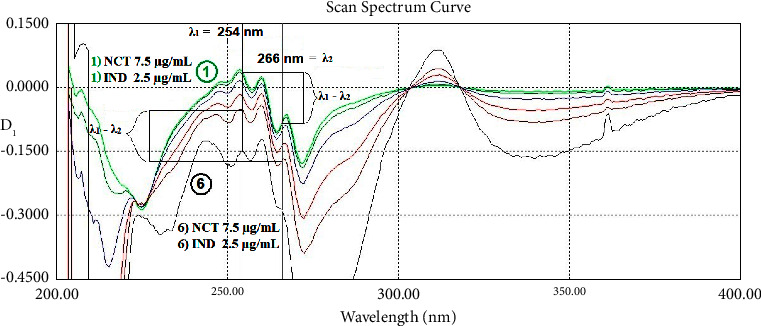
The first-order derivative spectra show a specificity test of different mixtures with 6 ratios containing constant nicotinamide 7.5 *μ*g/mL and series of indomethacin 2.5 (green), 3.75, 7.5, 15, 22.5, and 45 (black) *μ*g/mL at 254 and 266 nm as *λ*_1_ and *λ*_2_. All of them have the same value of *λ*_1_-*λ*_2_ except.

**Figure 11:**
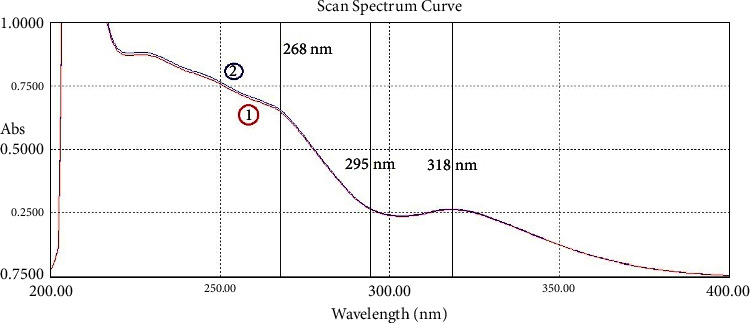
The resulting resolved spectrum (1) after SS method application for indomethacin of 15 µg/mL (red) compared to (2) the spectrum of the pure standard solution with the same concentration of IND (blue).

**Figure 12:**
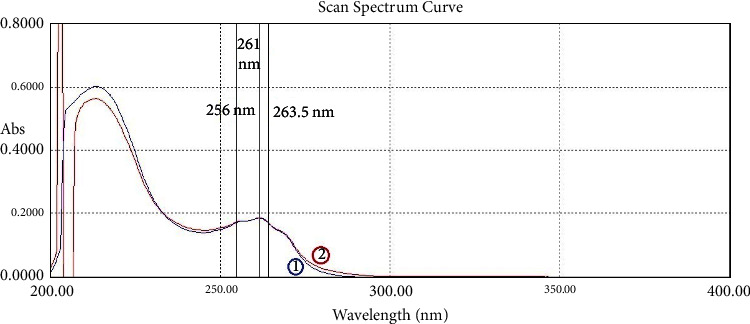
The resulting resolved spectrum (1) after RS application for nicotinamide of 7.5 µg/mL (blue) compared to (2) the spectrum of the pure standard solution with the same concentration of NCT (red).

Specificity studies were conducted on the mixture of IND and NCT with six ratios 1 : 3, 1 : 2, 1 : 1, 1 : 0.5, 1 : 0.33, and 1 : 0.17 (IND : NCT) using the three methods. For IND, a concentration of 15 *μ*g/mL was used for six concentrations of NCT, while for NCT, a concentration of 7.5 *μ*g/mL was used for six concentrations of IND. Results are shown in Table 4 for IND and Table 5 for NCT. For RS and SS, the SPF values were calculated and shown. The methods achieved accepted results in specificity tests for a wide range of the ratios of the binary mixture. Some methods for NCT determination, such as DWD_1_ and RS, failed the test only in the ratio of 1 : 0.17 (IND : NCT) because the absorption of the mixture exceeded the upper limit of the linearity range in the zero-order spectrum due to the high IND concentration of 45 *μ*g/mL along with 7.5 *μ*g/mL of NCT. In the next Figures 7–12, the spectra of the specificity test for IND and NCT are shown, where each of them shows the spectra of six mixtures of IND and NCT. Interchangeably, one of the two components has one constant concentration, while the other has six concentrations to obtain the six ratios of 1 : 3, 1 : 2, 1 : 1, 1 : 0.5, 1 : 0.33, and 1 : 0.17. ZCD_2_ is shown in Figure 7 for IND and Figure 8 for NCT. DWD_1_ is shown in Figure 9 for IND and Figure 10 for NCT. SS is shown in Figure 11 for IND, where the resolved spectrum of IND is shown with the standard one to be compared with. RS is shown in Figure 12 for NCT, where the resolved spectrum of NCT is shown with the standard one to be compared with.

#### 3.2.5. Application of the Proposed Method on Dosage Formulations

IR and SR were analyzed for their content of IND in addition to the content of the added NCT from standard solutions. Three ratios were analyzed for each dosage formulation and three repetitions for each ratio. In this application, the standard additions method has been used for both NCT and IND. All amounts and concentrations that were prepared for this test were mentioned in Sections 2.4.1 and 2.4.2. Data of the application on the dosage formulations are shown in Table 6. The results of ZCD_2_ were accepted in both dosage formulations in all ratios. The results of both DWD_1_ and RS were accepted in both dosage formulations, except for the ratio of 1 : 0.17.

#### 3.2.6. Comparing with Reference Methods

As there is no previous method for the simultaneous determination of IND and NCT in a mixture, each component was analyzed solely with the three proposed methods and a reference method to compare with. IND IR capsules were analyzed five times using the British pharmacopoeia (BP) HPLC assay method [24] in addition to the proposed methods. NCT pure powder (as there are no local NCT tablets available) was analyzed six times using the UV spectrophotometric assay method in BP [21] in addition to the proposed methods. The resulting data are shown in Table 7 along with statistical values such as standard deviation and variance of each method in addition to the *t*-test and *F*-test compared to the reference methods for each IND and NCT separately. The tests show that there are no statistically significant differences between the proposed methods and the reference ones.

## 4. Conclusion

The results of this work suggest that the developed methods are rapid, easy to use, accurate, precise, and simple. These techniques can be applied in regular quality control tests to simultaneously determine NCT and IND in binary mixtures, immediate-release capsules, and sustained-release capsules. These methods succeeded in determining both IND and NCT in the presence of excipients in the mixture without prior separation. The newly suggested co-amorphous systems have no special excipients or added materials other than the common ones in any oral dosage formulations. Therefore, these methods can be used to simultaneously determine IND and NCT in the co-amorphous systems achieving the same results above.

## Figures and Tables

**Table 1:** Analytical performance data for the proposed methods.

Parameter	Indomethacin			Nicotinamide		
Method	ZCD_2_	DWD_1_	SS	ZCD_2_	DWD_1_	RS
Wavelength (nm)	318.0	264.5–275.0	268.0	276.5	254.0–266.0	261.5
Linearity range (*μ*g/mL)	2.5–50.0	2.5–40.0	2.5–40.0	2.5–50.0	2.5–50.0	2.5–60.0
Determination coefficient *r*^2^	0.9998	0.9997	0.9997	0.9997	0.9999	0.9999
Slope	0.00324	0.00761	0.04292	0.01681	0.01602	0.02367
Intercept	0.00015	0.00179	0.00051	0.00029	0.00190	−0.00524
SD of slope	0.00002	0.00006	0.00032	0.00010	0.00007	0.00008
SD of intercept	0.00040	0.00127	0.00694	0.00268	0.00183	0.00255
95% confidence range of slope^*∗*^	(0.00321–0.00328)	(0.00747–0.00775)	(0.04215–0.04369)	(0.01658–0.01705)	(0.01586–0.01619)	(0.02349–0.02386)
95% confidence range of intercept^*∗*^	(−0.00081–0.00110)	(−0.00131–0.00489)	(−0.01646–0.01749)	(−0.00604–0.00663)	(−0.00243–0.00623)	(−0.01112–0.00064)
LOD (*μ*g/mL)	0.41	0.55	0.53	0.53	0.38	0.36
LOQ (*μ*g/mL)	1.25	1.66	1.62	1.59	1.14	1.08
System suitability^*∗∗*^	0.77	0.33	0.15	0.83	0.23	0.41

^*∗*^The range is represented by lower limit–upper limit. ^*∗∗*^RSD% of six repetitions of concentration of 20 *μ*g/mL for each of IND and NCT.

**Table 2:** Data of the accuracy tests of IND and NCT for the proposed methods.

Concentration (*μ*g/mL)	IND mean recovery ± RSD %^*∗*^			NCT mean recovery ± RSD %^*∗*^		
Method	ZCD_2_	DWD_1_	SS	ZCD_2_	DWD_1_	RS
5	99.06 ± 0.62	101.71 ± 0.45	99.44 ± 1.78	99.13 ± 1.75	100.15 ± 1.34	99.58 ± 1.41
10	98.79 ± 0.48	100.06 ± 1.44	99.44 ± 0.51	99.92 ± 0.26	99.21 ± 0.75	99.25 ± 1.14
15	99.93 ± 1.75	100.81 ± 1.11	101.59 ± 1.27	99.16 ± 1.53	98.20 ± 0.38	100.46 ± 0.49
20	100.35 ± 0.96	100.22 ± 0.98	100.09 ± 1.01	100.66 ± 0.62	100.46 ± 0.23	100.55 ± 1.52
25	99.78 ± 1.94	100.09 ± 1.66	99.99 ± 0.51	98.48 ± 1.87	100.44 ± 1.30	99.27 ± 0.34
30	99.70 ± 1.88	99.18 ± 0.81	98.98 ± 1.20	100.97 ± 0.55	100.71 ± 0.76	100.13 ± 0.81
Mean recovery ± RSD %^*∗∗*^	99.60 ± 1.94	100.35 ± 1.25	99.92 ± 1.28	99.72 ± 1.40	99.86 ± 1.17	99.87 ± 1.04

^*∗*^Three repetitions of each concentration were analyzed with nine total repetitions. ^*∗∗*^All 18 repetitions of each wavelength.

**Table 3:** Data of the precision study of IND and NCT for the proposed methods.

Precision	IND RSD%			NCT RSD%		
Method	ZCD_2_	DWD_1_	SS	ZCD_2_	DWD_1_	RS
Intraday day 1	1.49	0.97	0.35	1.21	0.89	0.61
Intermediate day 2	1.44	1.08	0.99	1.57	1.24	0.56
Intermediate day 3	1.37	1.23	1.09	1.66	1.28	0.63
Total repetitions^*∗*^	1.46	1.18	1.12	1.52	1.28	1.16

^*∗*^For each category, there are three concentrations and three repetitions for each concentration with a total of 27 repetitions.

**Table 4:** Data of the specificity test of IND for the proposed methods.

Ratio (IND : NCT)	NCT concentration (*μ*g/mL)	IND (15 *μ*g/mL) recovery%^*∗*^		
		ZCD_2_	DWD_1_	SS
1 : 3	45.0	100.90	100.68	99.09
1 : 2	30.0	100.07	100.30	98.13
1 : 1	15.0	99.65	99.64	99.18
1 : 0.5	7.5	98.61	99.14	98.08
1 : 0.33	5.0	101.53	99.60	98.73
1 : 0.17	2.5	99.24	100.65	100.46
SRF for SS method				0.9994

^*∗*^Mean recovery % of three experiments.

**Table 5:** Data of the specificity test of NCT for the proposed methods.

Ratio (IND : NCT)	IND concentration (*μ*g/mL)	NCT (7.5 *μ*g/mL) recovery%^*∗*^		
		ZCD_2_	DWD_1_	RS
1 : 3	2.50	101.80	100.52	101.41
1 : 2	3.75	101.40	98.19	101.79
1 : 1	7.50	98.12	98.10	99.42
1 : 0.5	15.00	98.44	98.60	98.99
1 : 0.33	22.50	98.28	100.19	98.39
1 : 0.17	45.00	101.96	114.41	93.86
SRF for RS method				0.9987

^*∗*^Mean recovery % of three experiments.

**Table 6:** Data of the analysis of IND and NCT in the dosage formulations with the proposed methods.

Dosage formulation + (IND : NCT) ratio	IND mean recovery ± RSD %^*∗*^			NCT mean recovery ± RSD %^*∗*^		
Method	ZCD_2_	DWD_1_	SS	ZCD_2_	DWD_1_	RS
IR^1^ mg (1 : 3)^*∗*^	100.42 ± 0.43	100.57 ± 0.72	101.02 ± 0.61	99.52 ± 1.46	100.92 ± 0.61	100.83 ± 0.38
IR (1 : 1)^*∗*^	101.28 ± 0.33	101.17 ± 0.64	101.14 ± 1.21	101.27 ± 0.34	99.45 ± 1.18	99.79 ± 0.34
IR (1 : 0.33)^*∗*^	101.36 ± 0.37	101.82 ± 0.59	99.98 ± 0.47	101.37 ± 1.18	100.62 ± 1.83	99.58 ± 1.14
IR mean recovery ± RSD %	101.02 ± 0.55	101.19 ± 0.78	100.71 ± 0.90	100.72 ± 1.30	100.33 ± 1.31	100.06 ± 0.85
SR^2^ (1 : 1)^*∗*^	98.63 ± 0.54	99.00 ± 0.50	99.16 ± 1.51	99.59 ± 1.44	99.91 ± 0.48	100.44 ± 0.65
SR (1 : 0.33)^*∗*^	99.63 ± 0.90	99.74 ± 0.48	99.51 ± 0.57	101.26 ± 0.95	101.62 ± 0.34	98.90 ± 0.71
SR (1 : 0.17)^*∗*^	98.62 ± 0.87	103.73 ± 0.34	98.63 ± 0.66	101.53 ± 0.89	104.53 ± 0.77	96.79 ± 0.97
SR mean recovery ± RSD %	98.96 ± 0.85	100.82 ± 2.22	99.10 ± 0.95	100.79 ± 1.32	102.02 ± 2.04	98.71 ± 1.75

^1^IR contains 50 mg of IND as labeled. ^2^SR contains 75 mg of IND as labeled. ^*∗*^Three repetitions of each ratio.

**Table 7:** Data of the comparison between the proposed methods and reference methods.

	Sample number	Indomethacin in IR capsules^1^				Pure nicotinamide^2^			
		ZCD_2_	DWD_1_	SS	^*∗*^BP19 HPLC	ZCD_2_	DWD_1_	RS	^*∗*^BP19 UV
Recovery%	1	100.04	100.58	101.32	99.55	100.21	100.23	99.13	100.14
	2	101.09	100.61	101.22	98.74	99.02	99.95	98.96	99.97
	3	101.04	99.79	100.89	100.21	98.04	99.82	98.85	99.86
	4	101.27	100.86	100.15	100.80	98.10	99.73	98.53	99.53
	5	101.46	101.25	100.42	101.12	98.55	99.54	100.30	99.30
	6	—	—	—	—	98.34	99.82	98.07	99.06
Mean recovery%		100.98	100.62	100.80	100.08	98.71	99.85	98.97	99.64
SD		0.5509	0.5350	0.5054	0.9609	0.8160	0.2309	0.7500	0.4166
Standard error		0.2464	0.2393	0.2260	0.4297	0.3332	0.0943	0.3062	0.1701
Variance		0.3034	0.2863	0.2554	0.9233	0.6659	0.0533	0.5625	0.1736
*t*-test with ref method ^*∗∗*^		1.809	1.086	1.475	—	2.490	1.064	1.908	—
*F*-test with ref method ^*∗∗∗*^		3.043	3.225	3.615	—	3.837	3.254	3.241	—

^1^The number of samples of immediate-release capsules was 5 (*n* = 5). ^2^The number of samples of pure nicotinamide was 6 (*n* = 6). ^*∗*^The reference methods to be compared with. ^*∗∗*^Critical values of the two-tailed test in 5% significance level are 2.776 for *n* = 5 and 2.571 for *n* = 6. ^*∗∗∗*^Critical values of the *F*-test in 5% significance level are 6.39 for *n* = 5 and 5.05 for *n* = 6.

## Data Availability

All data, figures, and tables are available within the manuscript.
